# Genomic Changes and Genetic Divergence of *Vibrio alginolyticus* Under Phage Infection Stress Revealed by Whole-Genome Sequencing and Resequencing

**DOI:** 10.3389/fmicb.2021.710262

**Published:** 2021-10-04

**Authors:** Wenjie Zhou, Yingying Li, Zhuobo Li, Bo Ma, Xiao Jiang, Chaoqun Hu, Yongxing Ai, Peng Luo

**Affiliations:** ^1^College of Animal Science, Jilin University, Changchun, China; ^2^CAS Key Laboratory of Tropical Marine Bio-Resources and Ecology (LMB), Guangdong Provincial Key Laboratory of Applied Marine Biology (LAMB), South China Sea Institute of Oceanology, Chinese Academy of Sciences, Guangzhou, China; ^3^Southern Marine Science and Engineering Guangdong Laboratory (Guangzhou), Guangzhou, China; ^4^Geological Survey Institute of Guangzhou, Guangzhou, China; ^5^University of Chinese Academy of Sciences, Beijing, China

**Keywords:** *Vibrio alginolyticus*, phages, genetic divergence, phage-resistant mutations, genomic changes

## Abstract

Bacteriophages (phages) and their bacterial hosts were the most abundant and genetically highly diverse organisms on the earth. In this study, a series of phage-resistant mutant (PRM) strains derived from *Vibrio alginolyticus* were isolated and Infrequent-restriction-site PCR (IRS-PCR) was used to investigate the genetic diversity of the PRM strains. Phenotypic variations of eight PRM strains were analyzed using profiles of utilizing carbon sources and chemical sensitivity. Genetic variations of eight PRM strains and coevolved *V. alginolyticus* populations with phages were analyzed by whole-genome sequencing and resequencing, respectively. The results indicated that eight genetically discrepant PRM stains exhibited abundant and abundant phenotypic variations. Eight PRM strains and coevolved *V. alginolyticus* populations (VE1, VE2, and VE3) contained numerous single nucleotide variations (SNVs) and insertions/indels (InDels) and exhibited obvious genetic divergence. Most of the SNVs and InDels in coding genes were related to the synthesis of flagellar, extracellular polysaccharide (EPS), which often served as the receptors of phage invasion. The PRM strains and the coevolved cell populations also contained frequent mutations in tRNA and rRNA genes. Two out of three coevolved populations (VE1 and VE2) contained a large mutation segment severely deconstructing gene *nrdA*, which was predictably responsible for the booming of mutation rate in the genome. In summary, numerous mutations and genetic divergence were detected in the genomes of *V. alginolyticus* PRM strains and in coevolved cell populations of *V. alginolyticus* under phage infection stress. The phage infection stress may provide an important force driving genomic evolution of *V. alginolyticus*.

## Introduction

Bacteriophages (phages) and their bacterial hosts are the most abundant and genetically highly diverse organisms on the earth (Suttle, 2005; Weitz et al., 2013; Batinovic et al., 2019). Phages propagate through hijacking replication and metabolic mechanisms of bacterial hosts and induce the lysis of bacterial hosts, and thus bring a very strong selection stress for bacterial survival and evolution (Thurber, 2009; Gómez and Buckling, 2013; Warwick-Dugdale et al., 2019), which results in the occurrence of phage-resistant mutant (PRM) population. For a long time, bacteria–phage interaction is believed as an important driver of ecological and evolutionary processes for both communities (Fuhrman, 1999; Koskella and Brockhurst, 2014; Shabbir et al., 2016; Hampton et al., 2020), however, studies on genetic variations of PRM strains and genomic evolution of coevolved bacterial populations with phages are very rare. Some studies on comparative genomics between PRM and ancestor strains revealed some genetic mutations, mainly focusing on the effect of mutations on the expression of genes related to cell surface structure, such as LPS and flagella (Kashiwagi and Yomo, 2011; Castillo et al., 2015; Lis and Connerton, 2016; Gonzalez et al., 2018). Little is known about detailed genetic variations of PRM strains on whole-genomic scale under phage infection stress. Scanlan et al. (2015) first revealed that coevolution with phage Phi2 drives genomic-wide host evolution and constrains the acquisition of abiotic-beneficial mutations in *Pseudomonas fluorescens* through whole-genome sequencing, and the isolates with discrepant phenotypes in coevolved populations exhibited much more numerous mutations and much higher genetic divergence compared to these in evolved populations (Scanlan et al., 2015). Resequencing provides a new approach to probe into the genetic variations and mutation frequencies within cell populations. However, so far as now, the technique was only used to investigate occurrence frequencies of mutation sites from the perspective of phage φ2 when it was coevolved with its host *P. fluorescens* (Paterson et al., 2010).

Phages are the most abundant viruses in ocean, and it is estimated that phages kill and lyse between 15 and 40% of ocean’s bacteria every day, which poses a huge pressure on the survival of marine bacteria (Danovaro et al., 2011). Ocean contains the most abundant bacteria and phages on the Earth, however, the research on the genomic evolution of marine bacteria under phage infection stress has so far been completely blank. *Vibrio alginolyticus* is one of the most common bacterial species in estuary and marine environments (Luo et al., 2012; Fu et al., 2016), and it is also a conditional pathogen for human beings (Daniels and Shafaie, 2000; Slifka et al., 2017) and many kinds of marine fish, shellfish, and crustaceans (Pruzzo et al., 2006; Abdelaziza et al., 2017). In this study, we first isolated numerous PRM strains derived from an ancestor strain *V. alginolyticus* E06333 under phage infection stress. The whole genomic sequences of several genetically divergent mutants were analyzed focusing on single nucleotide variations (SNVs) and insertions/deletions (InDels) and their potential functions. Besides, resequencing of the evolved populations of *V. alginolyticus* E06333 and the coevolved populations with enriched phages at different periods were carried out to characterize mutation sites and their frequencies of occurrence.

## Results

### Screening of Phage-Resistant Mutant Strains by Double-Layer Plate Method

**FIGURE 1:**
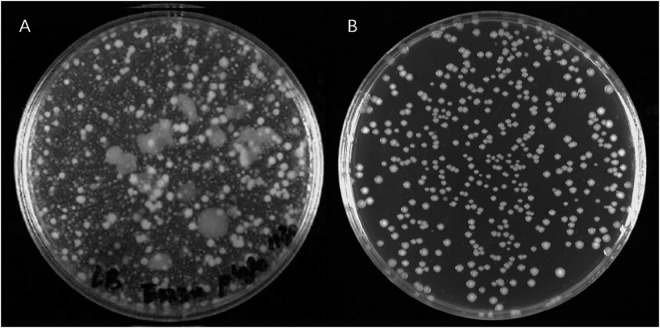
The phage resistant colonies derived from *V. alginolyticus* E06333 and normal colonies *V. alginolyticus* E06333. **(A)** The phage resistant colonies occurred on a double-layer plate with enriched lytic phages. **(B)** The normal colonies of *V. alginolyticus* E06333 on a LB plate.

A large number of PRM colonies derived from *V. alginolyticus* E06333 appeared on double-layer plates, and the sizes and surface morphologies of PRM colonies were very different from these grown normally on LB plates without phages (Figure 1), which implied that genetic variations harbored among these PRM colonies. Totally 80 PRM strains of *V. alginolyticus* were acquired.

### Infrequent-Restriction-Site-PCR Exhibited Extensive Genetic Variations in the Phage-Resistant Mutant Strains

**FIGURE 2:**
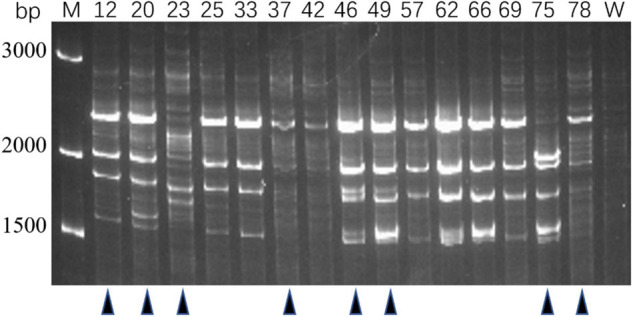
Genotyping of typical PRM strains derived from *V. alginolyticus* E06333 by IRS-PCR. These PRM strains exhibit obviously different fingerprints from the primary *V. alginolyticus* E06333. The numbers on the top represent the strain numbers. The black triangles on the bottom present eight PRM strains that are further selected for subsequent analysis. Eight PRM strains were renamed (according to the order from left to right) as VAM01, VAM02, VAM03, VAM04, VAM05, VAM06, VAM07, and VAM08, respectively.

Genotypes of 80 PRM strains were determined by IRS-PCR, and the results indicated most PRM strains did not show obvious genetic variations in this method except that 15 PRM strains generated obvious discrepant and bright bands within the range of 1500–3000 bp (Figure 2). Among them, 8 PRM strains were singled out for subsequent experiments, and they were renamed as VAM01–VAM08 (Figure 2).

### Phenotypic Variations of the Phage-Resistant Mutant Strains Revealed by Biolog Assays

**FIGURE 3:**
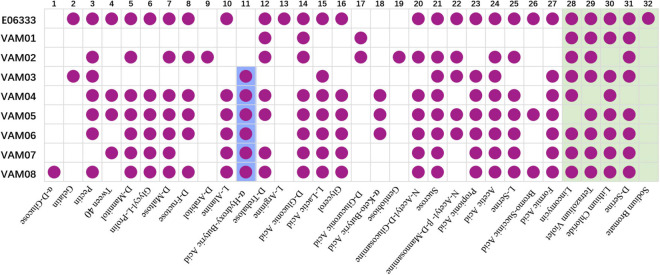
Biochemical profiles of eight PRM strains and the primary strain E06333 from *V. alginolyticus* revealed by Biolog assays. The purple circles in boxes represent a positive reaction (growth). The purple circles with blue background represent six PRM strains obtained the ability of utilizing α-Hydroxy-Butyric Acid. The boxes with light green background represent five tests of chemical sensitivity.

Biolog assays with GEN III microplates indicated that eight PRM strains generated 32 differentiated reactions compared with the ancestor strain E06333, among which 27 reactions were related to carbon utilization and 5 reactions were related to chemical sensitivity (Figure 3). Each PRM strain had a unique reaction profile, but all PRM strains tended to lose some phenotypes rather than to acquire some phenotypes (Figure 3). Among them, PRM strain VAM01 lost the most 19 positive phenotypes of utilizing 19 carbon sources when compared with wild strain *V. alginolyticus* E06333. All the PRM strains lost the ability to utilize L-Arginine and became sensitive to sodium bromate. Eight PRM strains only shifted to acquire 1–3 positive phenotypes of utilizing carbon sources, and 6 out of 8 PRM strains obtained the ability utilizing α-hydroxy-butyric Acid (Figure 3). Biolog assays clearly indicated that eight PRM strains generated rich and obvious phenotypic changes and these changes were not significantly associated with anti-phage infection, which suggested the genetic variations harbored in these PRM strains.

### Discrepant Mutation Sites and Their Related Genes in the Phage-Resistant Mutant Strains of *V. alginolyticus*

**FIGURE 4:**
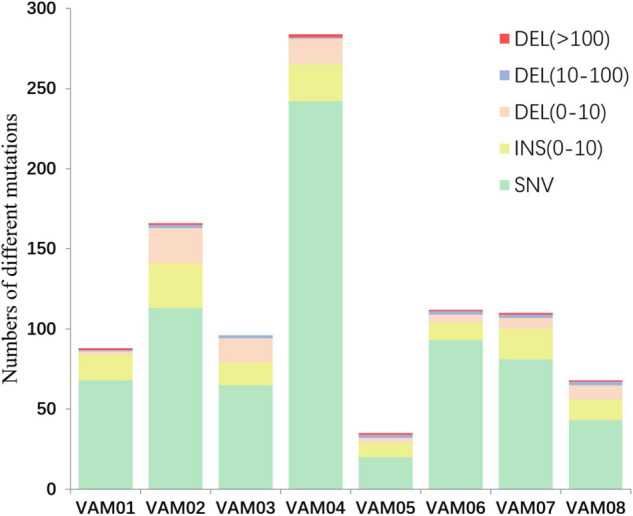
The numbers of different mutations in eight PRM strains from *V. alginolyticus*. The mutations were classed into insertion (INS), deletions (DEL), and single nucleotide variations (SNVs) according to the mutation types and sizes. An 11-bp insertion in the PRM strain VAM03 was not shown in this figure.

Comparative genomics of *V. alginolyticus* E06333 and eight PRM strains revealed numerous SNVs and InDels in eight PRM strains. Eight PRM strains had discrepant numbers of SNVs and InDels with different sizes (Figure 4). The PRM strain VAM04 harbored the most SNVs and InDels including 242 SNVs and 42 InDels while the PRM strain VAM05 harbored the least SNVs and InDels including 20 SNVs and 15 InDels (Figure 4). The PRM strain VAM03 harbored an only >10-bp insertion (11 bp), and each PRM strain contained 1 or 2 big deletions that exceeded 100 bp (actually >400 bp) except for the strain VAM03 (Figure 4).

Further identification of these numerous mutation sites revealed that 15 mutation sites occurred within coding genes of eight PRM strains, and these intragenic mutation sites included 11 scattered non-synonymous SNVs, 3 InDels and one large region containing concentrated SNVs, LCM1 (Table 1), wherein 8 PRMs had discrepant SNV distribution. The PRM strains, VAM01–VAM08, contained 3, 5, 7, 7, 4, 8, 4, and 4 SNVs and InDels within coding genes, respectively, and each PRM strain had a unique mutation profile (Table 1). 15 mutation sites covered 12 genes involving in different functions, including secretion of lectins and toxins, bacterial outer membrane translocation and assembly, polysaccharide synthesis, flagella synthesis and regulation, and aerotolerance operon (Table 1). Among 12 genes, the mutations of *fliF*, *flhA*, *fleQ*, *epsD*, *ugdH*, *glyT*, and *ytfM* were predictably related to the phage resistance of the PRM strains in varying degrees (Table 1), and they participated in the synthesis of flagellar, extracellular polysaccharide (EPS) including capsular polysaccharides and lipopolysaccharides (LPS). The mutations in *flhA* and *fliF* were non-sense mutations, leading to the premature termination of peptide synthesis. A 11-bp insertion gave rise to the change of 24 amino acids residues at the end of the *espD* gene. The SNVs at nt2573434 and nt2573439 resulted the changes of two amino acids residues (P/L and I/Y) in the translated protein, UGDH, coded by *ugdH*, two changed amino acids located on conserved sites of NADB Rossmann Superfamily. A 18-bp deletion in *batB* of aerotolerance operon was the only mutation region shared by eight PRM strains. The end of *batB* contained 55 core repeats of “GCAACA,” and the deletion contained three core repeats that resulted in truncated and altered amino acids in predicted gene translocation. Six PRM strains (VAM02, VAM03, VAM04, VAM06, VAM07, and VAM08) contained SNVs in *omp1* coding for a putative calcium-binding outer membrane-like protein in T1SS system. Four strains (VAM02, VAM04, VAM06, and VAM07) and 6 PRM strains (VAM01, VAM03, VAM04, VAM05, VAM06, and VAM08) harbored large fragment mutations in *rtx1*, and *rtx2*, respectively. The gene *rtx1* encoded for a tandem-95 repeat protein containing a cadherin-like domain, and the gene *rtx2* encoded a putative RTX toxin in T1SS system. Three genes, *omp1, rtx1*, and *rtx2*, were featured by many tandem repeats and large size (>10000 bp). Besides, it is little known about the function of a gene, *hyp1*, in *V. alginolyticus* and SNVs in *batB* were predicted not to have direct relations to phage resistance.

**TABLE 1:** Mutation sites in coding genes of eight PRM strains derived from *V. alginolyticus* E06333.

**Mutation sites**	**Variation; AA change**	**Gene**	**Protein encoded by gene**	**Protein function**	**1**	**2**	**3**	**4**	**5**	**6**	**7**	**8**
498716	T/C; L/S	*rtX2*	RTX toxin in T1SS	toxin	√		√	√	√	√		√
731572	G/T; T/S	*Omp1*	putative calcium-binding outer membrane-like protein	agglutinin		√	√	√		√	√	√
731573	C/G; T/S	*Omp1*	putative calcium-binding outer membrane-like protein	agglutinin		√	√	√		√	√	√
737614	C/G; A/P	*Omp1*	putative calcium-binding outer membrane-like protein	agglutinin						√		
758419–759137	LCM1	*rtX1*	tandem-95 repeat protein containing a cadherin-like domain	unknown		√		√		√	√	
1406251	C/A; E/*	*flhA*	polar flagellar biosynthesis protein	flagellar		√						
1421835	A/C; L/*	*fliF* ^1^	polar flagellar M-ring protein	flagellar	√							
1425623	G/A; V/A	*fleQ*	flagellar master regulator	flagellar			√					
2558866	11-bp insertion	*epsD*	glycosyl transferase domain protein	EPS synthesis			√					
2564097	A/-; frame shift	*glyT*	glycosyl transferase	EPS synthesis			√					
2573434	C/T; P/L	*ugdH*	UDP-glucose dehydrogenase	LPS synthesis				√		√		
2573439	G/T; I/Y	*ugdH*	UDP-glucose dehydrogenase	LPS synthesis					√			
2625978	G/A; A/T	*ytfM*	uncharacterized protein YtfM precursor	outer membrane				√		√		
91359–91376 (chr2)	18-bp deletion; frame shift	*batB*	TPR domain protein in aerotolerance operon	aerotolerance operon	√	√	√	√	√	√	√	√
444588 (chr2)	C/A; A/D	*hyp1*	hypothetical protein	unknown					√			

*LCM1 represents one large region containing concentrated SNVs, wherein 8 PRM strains had different SNV distribution. “1–8” at the right columns represent eight PRM strains of *V. alginolyticus*, VAM01, VAM02, VAM03, VAM04, VAM05, VAM06, VAM07, and VAM08, respectively. “√” represent one strain has a corresponding mutation site.*

“*” represents a stop codon.

### Mutations in DNA Regions Related to Non-coding RNA Genes of the Phage-Resistant Mutant Strains

It is notable that eight PRM strains also harbored 12 SNVs and InDels in non-coding RNA genes (Table 2), including 5 big deletion regions (422–677 bp) and 3 concentrated SNV regions (SNVs > 5 within the range of 400 bp), and 4 scattered SNV regions, respectively (Table 2). Five big deletion regions all occurred in the tandem repeat regions of tRNA genes. In addition, 2 concentrated SNV regions and 4 SNVs occurred in the 16S or 23S rRNA genes. Each PRM strain had a different mutation profile in non-coding RNA genes, which further exhibited genetic divergence among eight PRM strains.

**TABLE 2:** Mutation sites in non-coding RNA genes of eight PRM strains derived from *V. alginolyticus* E06333.

**Position (nt)**	**Variations**	**RNA genes**	**PRM strains**							
			**1**	**2**	**3**	**4**	**5**	**6**	**7**	**8**
12883–13390	508-bp deletion	3 tRNA genes			√	√	√	√	√	√
12883–13559	677-bp deletion	4 tRNA genes	√	√						
1733939–1734456	518-bp deletion	4 tRNA genes				√				
1828237	SNV (T-C)	16S rRNA gene	√						√	
1938591	SNV (T-C)	16S rRNA gene					√			
2040854–2041275	422-bp deletion	4 tRNA genes		√	√	√	√	√	√	√
2167079	SNV (T-A)	16S rRNA gene	√		√				√	
2271277–2271509	2 SNVs in 362 bp	16S rRNA gene			√	√				
2354021	SNV (G-A)	16S rRNA gene	√	√						
2358245–2358420	21 SNVs in 176 bp	23S rRNA		√						√
3087203–3088807	473-bp deletion	4 tRNA genes	√	√						
3088235–3088555	35 SNVs in 321 bp	4 tRNA genes			√	√				

### Divergent Evolution of Coevolved *V. alginolyticus* E06333 Cell Populations Revealed by Genomic Resequencing

**FIGURE 5:**
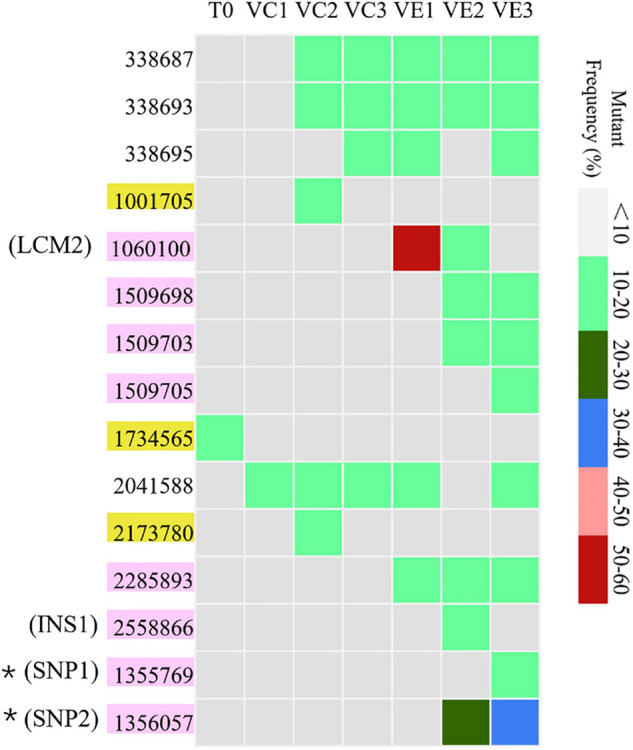
The distribution and the mutation frequencies of SNVs and InDels in evolved and coevolved *V. alginolyticus* cell populations revealed by resequencing. T0: the collected sample before grouping at the early exponential stage (*OD*_600 nm_ = 0.6–1); VC1: collected samples from evolved cell populations at 2 h; VC2: collected samples from evolved cell populations at 13 h; VC3: collected samples from evolved cell populations at 22 h; VE1: collected samples from coevolved cell populations at 2 h; VE2: collected samples from coevolved cell populations at 13 h; VE3: collected samples from coevolved cell populations at 22 h. Mutation sites with yellow background represent 2 unique SNVs in the evolved cell populations. Positions of mutation sites with purple background represent 8 unique mutations in the coevolved cell populations. Mutation frequencies of these sites are shown with different colors.

Resequencing facilitates to reveal of the frequencies of mutations in each bacterial cell population. The SNVs and InDels detected by resequencing data were shown in Supplementary Table 1. Totally, 14 SNVs and InDels were detected in the evolved cell populations (VC1, VC2, and VC3) and the coevolved cell populations (VE1, VE2, and VE3), including 4 SNVs, 10 InDels, and 1 large region of 1260 bp containing concentrated point mutations (LCM2) (Figure 5). Coevolved cell populations of *V. alginolyticus* contained 8 unique SNVs and InDels (53% of total numbers) while evolved cell populations only had 2 unique SNVs (13% of total numbers of mutation sites), which manifested that most of mutation sites occurred in coevolved cell populations with phages. It clearly indicated that coexisting phages markedly raised detectable SNVs and InDels in *V. alginolyticus* cell populations. Each coevolved cell populations at different time points (VE1 at 2 h, VE2 at 13 h, VE3 at 22 h) had a unique mutation profile, and they were also very different from the mutation profiles of evolved cell populations (Figure 5). The result manifested that phage infection stress drove and accelerated the divergent evolution of *V. alginolyticus* cell populations.

### Unique Mutation Sites in Coevolved Cell Populations of *V. alginolyticus* and Their Functions of Related Genes

Though the evolved cell populations harbored 2 unique SNVs and 4 shared SNVs (Figure 5), here we only focused on the unique mutation sites in coevolved cell populations of *V. alginolyticus* and their predicted functions of related genes (Table 3). Among 8 unique mutation sites in the coevolved cell populations of *V. alginolyticus*, 3 SNVs occurred in *tsaA* coding for a tRNA (Thr-GGU) A37N-methylase, and one InDel occurred in the upstream of *glyS* coding for a Glycyl-tRNA synthetase β chain, and therefore these four mutation sites are closely related to tRNA synthesis. An insertion of 11 bp (INS1) at nt2558866 resulted in a frameshift mutation of *epsD*, and this 11-bp insertion also occurred in the PRM strain VAM03 (Table 1). The insertion gave rise to the change of 24 amino acids residues at the end. The gene *epsD* is predicted related to the synthesis of extracellular polysaccharides and catalyze the transfer of UDP-glucose or UDP-galactose to lipid carriers, which is the first step in the synthesis of oligosaccharides. The coevolved cell populations also contained 2 SNVs (SNP1 and SNP2) in *galE*, and SNP2 caused the premature termination of transcription of *galE*. The large mutation region of 1260 bp (LCM2) in VE1 and VE2 populations severely deconstructed gene *nrdA*, which blocked the synthesis of ribonucleotide reductase (RNR). The mutation frequency of this gene at the stage of VE1 reached at as high as 55% and then dramatically declined to 13% at the stage of VE2 and below detection limitation (<10%) at the stage of VE3.

**TABLE 3:** Mutant sites and related genes in evolved and coevolved *V. alginolyticus* cell populations.

**Position**	**Variation**	**Mutation description**	**Genes**	**Protein name**	**Protein function**	**T0**	**VC1**	**VC2**	**VC3**	**VE1**	**VE2**	**VE3**
338687	C/−	frameshift deletion	*cheC*	CheC	Flagella rotation			√	√	√	√	√
338693	C/−	frameshift deletion	*cheC*	CheC	Flagella rotation			√	√	√	√	√
338695	GCTT/−	frameshift deletion	*cheC*	CheC	Flagella rotation				√	√		√
1001705	TTT/−	non-frameshift deletion	*ttdA*	Fumarate hydratase class I, aerobic	Oxaloacetate reduction			√				
1060100–1061359	LCM2	stopgain	*nrdA*	Ribonucleotide reductase, α subunit	DNA replication and repair					√	√	
1509698	−/G	frameshift insertion	*tsaA*	tRNA (Thr-GGU) A37N*-*methylase	tRNA synthesis						√	√
1509703	A/−	frameshift deletion	*tsaA*	tRNA (Thr-GGU) A37 N-methylase	tRNA synthesis						√	√
1509705	GGGA/−	frameshift deletion	*tsaA*	tRNA (Thr-GGU) A37 N-methylase	tRNA synthesis							√
1734565	A/G	intergenic mutation	–	tRNA-Arg-ACG, tRNA-Arg-ACG	tRNA synthesis	√						
2041588	G/A	intergenic mutation	–	tRNA-Gly-GCC, tRNA-Met-CAT	tRNA synthesis		√	√	√	√		√
2173780	−/C	frameshift insertion	–	tRNA methyltransferase	RNA methylation			√				
2285893	AT/-	upstream	*glyS*	Glycyl-tRNA synthetase β chain	Glycyl-tRNA synthesis					√	√	√
2558866	−/11-bp insertion	frameshift insertion	*epsD*	Glycosyl transferase domain protein	EPS synthesis						√	
1355769 (chr2)	T/A (SNP1)	missense mutation	*galE*	UDP-glucose 4-epimerase	LPS synthesis							√
1356057 (chr2)	C/T (SNP2)	stopgain	*galE*	UDP-glucose 4-epimerase	LPS synthesis						√	√

*LCM2 represents a large region of 1260 bp containing concentrated point mutations (LCM2). “√” represent one certain population of *V. alginolyticus* contained one detected mutation site.*

## Discussion

Exploring the adaptive evolution of environmental microbes represents an increasing interest under the background of the extensively used high-through technologies, which facilitates us to understand the history of bacterial genomic evolution and bacterial genetic diversity. The arms race between bacteria and their phage has been long believed to be an important force for mutual evolution (Hampton et al., 2020; Safari et al., 2020), but few details in terms of bacterial whole-genome scale have been revealed. In three PRM strains, three variant genes related to flagella synthesis and regulation, *flhA, fliF, and fleQ*, were detected, among which the SNVs in *flhA* and *fli*F resulted in the premature termination of flagella synthesis. Flagella are often used as a receptor for phages to invade bacterial hosts (Kropinski et al., 2012; Choi et al., 2013; Silva et al., 2016), and therefore the inhibition of flagellin synthesis in the PRM strains may affect the phage adsorption process. Besides, the PRM strains displayed various mutations in *ugdH* and *epsD*, and coevolved *V. alginolyticus* populations harbored unique mutations in *galE* and *epsD*. The product of *galE*, GalE, was an important enzyme for the synthesis of active carbohydrates. It was frequently shared in a variety of EPS biosynthetic pathways (Bahat-Samet et al., 2004; Schaper et al., 2019) and affected the biosynthesis of a variety of bacterial lipopolysaccharides (Bengoechea et al., 2002). Four genes involved in the synthesis of extracellular polysaccharides including capsular polysaccharides and lipopolysaccharides were thought to be responsible for phage adsorption (Baldvinsson et al., 2014; Dang and Lovell, 2015; Ha et al., 2019). Mutations in the genes encoding for flagellin synthesis and extracellular polysaccharides were often reported in phage-resistant mutants since these cell structures served as the receptors of phages (Nesper et al., 2001; Gonzalez et al., 2018; Cai et al., 2019; Schaper et al., 2019). From this regard, generating mutations in the genes encoding for flagellin synthesis and extracellular polysaccharides is likely a common strategy to cope with phage infection in *V. alginolyticus*. Reduced virulence was often observed in phage-resistance mutants as mutant genes changed surface antigens that are crucial for both phage adsorption and cellular invasiveness (Laanto et al., 2012; Castillo et al., 2015; Sumrall et al., 2019; Markwitz et al., 2021). Except these virulent genes involving in phage adsorption generated mutations, a virulence-associated gene *rtx2* coding for RTX toxin in T1SS also generated mutations. Therefore, mutations under phage infection stress are not limited within genes responsible for phage adsorption. Besides, the functions of some genes bearing mutations in the PRM strains and in the coevolved cell populations remain unknown, and it is worthy to explore their potential roles in phage infections in future.

It is generally recognized that rRNA genes unlikely generate rapid and frequent changes as they perform the most fundamental and important functions in life. Therefore, the rRNA genes were once considered the ideal chronometer to record the evolutionary history of life, potentially all the way back to the last common ancestor (Woese, 1987, 1998), and rRNA sequence comparisons led to the construction of a “universal tree of life,” dividing all life on the Earth into three equidistant domains: eukarya, bacteria, and archaea (Woese et al., 1990). Until now, 16S and 23S rRNA gene mutations were often observed in some antibiotic-resistant bacterial strains (Bachir et al., 2018; Chen et al., 2018; Lauener et al., 2019), wherein antibiotics generally impact the normal functions of 16S and 23S rRNA or their coupling ribosomes. It is obvious that the mutations of 16S and 23S rRNA genes have not contributed to bacterial resistance against phage. This study first demonstrated that phage infection stress can change the sequences of highly conserved rRNA genes, which complicate bacterial phylogenetics based on rRNA genes, and thus phage infection stress has a possibility to drive bacterial rRNA molecular evolution. In addition, few mutations in tRNA genes or related DNA regions were noticed in bacteria. However, it’s surprising that frequent mutations of tRNA genes were also observed in eight PRM strains. The rRNA- and tRNA-related mutations in PRM strains of *V. alginolyticus* under experimental conditions urge us to speculate that similar incidents may occur with a certain frequency in natural bacterial communities due to the prevalence of phages and strong selective stress by them. In this study, we observed the reduced copy numbers of tRNA genes (Table 2). For instance, the chromosomal site (Chr1: nt12887–13817) of the primary strain E06333 contains six tRNA-Tyr genes, but in the PRM VAM01 and VAM02 only remained two tRNA genes. By Blast, we found that conspecific strains not only vary copy numbers of tRNA genes [e.g., the strains VIO5 (AP022861.1) and YM19 (AP022863.1)] at this chromosomal site but also contained variable sequences. Yona et al. (2013) first carried out a systematic search in hundreds of genomes that revealed tRNA gene mutations occur throughout the tree of life. They further demonstrate that mutation in tRNA genes is a common adaptive mechanism when meeting new translational demands (Yona et al., 2013). Until now, there are no evidences indicating direct correlation between phage-resistant mechanisms and rRNA and tRNA gene mutations. Hence, we speculated the variations in tRNA pool as an adaptive mechanism to meet the new translational demands in the PRM strains. Together, these results indicated that phage infection stress can drive bacterial rRNA and tRNA gene evolution.

It is notable that variations in coding genes and rRNA- and tRNA- genes among these PRM strains lacked significant correlation with the phenotypical shifts revealed by Biolog assays. Therefore, it is reasonable to speculate some genetic changes affecting phenotypic characteristics were not discovered. On the other hand, though many mutation sites were detected in the draft genomes of eight PRM strains, the gaps between scaffolds potentially contained some mutation sites not detected. As a result, the actual numbers of SNVs and InDels may exceed our calculation. Numerous genetic variations in the PRM strains and the coevolved populations also raised a question whether phage infection stress induced the generation of most observed mutations in *V. alginolyticus* and boost the mutation rate. Through whole-genome sequencing, it is estimated that spontaneous mutations in *Escherichia coli*, as a typical representation of prokaryotes is 1.0 × 10^–3^ mutations per genome per generation (Lee et al., 2016). According to this spontaneous mutation rate, it is impossible to accumulate numerous genetic variations in the genomes of the PRM strains and coevolved populations of *V. alginolyticus* even at the longest generation time of 22 h (about 66 generations) for VE3 populations. Therefore, increased genetic variations (mutations) in the PRM strains and coevolved populations were not generated by spontaneous mutations but mainly induced by phage infection stress, which provides one important force driving genomic evolution of *V. alginolyticus* and accelerate genetic divergence via boosting mutation rate. Swings et al. (2017) found that mutation rates of *E. coli* populations rise when cells experience higher stress and decline again once cells are adapted, and they identified cellular mortality as the major force driving the quick evolution of mutation rates (Swings et al., 2017). Increased mutation rates under environmental stress were also observed in some cases (Bjedov, 2003; Galhardo et al., 2007; MacLean et al., 2013). All these findings strongly supported the idea that mutation rate variability plays a key role in adaptive evolution under selective pressure (Engelhardt and Shakhnovich, 2019). Together, these studies suggested that the role of environmental stress is not limited to naturally select preexisting mutants and it is not the whole story that all mutations are random and evolution is slow. Therefore, it shed some light on the acknowledges of microbial genomic evolution in a natural environment full of competition and adverseness.

Interestingly, the coevolved populations VE1 were observed to have a high-frequency (53%) large mismatch mutation in *nrdA* coding for a ribonucleotide reductase (RNR) alpha subunit, RNR1. RNR catalyzes the conversion of nucleoside diphosphates (NDPs) into deoxynucleotides and is a key enzyme for DNA replication and repair in all organisms (Cotruvo and Stubbe, 2008). The levels of the cellular dNTPs are tightly controlled, in large part through allosteric control of RNR (Ahluwalia et al., 2012; Zhu et al., 2017). Ahluwalia et al. (2012) found that a set of RNR1 mutants of *E. coli* changed dNTP pools and the mutation rate of bacteria also increases with the increase of the dNTP change range. They further found that even relatively modest dNTP pool deviations caused by one set of RNR1 mutants gave rise to exceptionally strong mutator phenotypes (>1,000-fold increases) (Ahluwalia et al., 2012). From this case, we speculated that high-frequency and large mutation in *nrdA* likely changed the dNTPs pool in *V. alginolyticus* and mainly contribute to the booming of the mutations. The mutation frequency of *nrdA* dramatically declined to 13% and below the detection limit (<10%). This sharp decline in coevolved populations was likely due to the adaptive evolution of *V. alginolyticus* under phage stress at different stages. Once numerous phage-resistant mutator phenotypes merged, it is necessary to regain the normal intracellular environment to ensure accurate DNA replication and stable propagation. Therefore, we speculated that the occurrence of PRM strains containing numerous mutation sites is likely the consequence of the dynamic change of *nrdA* in a certain stage though the PRM strains did not harbor *nrdA* mutation. The functions of RNR in *V. alginolyticus* need to be further explored in future.

Bacteria and phages are locked in a constant arm race and both are perpetually changing their tactics to overcome each other (Hampton et al., 2020; Safari et al., 2020). Bacteria use various strategies to overcome the invading phages, including adsorption inhibition, restriction-modification (R/E) systems, CRISPR–Cas (clustered regularly interspaced short palindromic repeats–CRISPR-associated proteins) systems, abortive infection (Abi), etc (Safari et al., 2020). To counteract, phages employ intelligent tactics for the nullification of bacterial defense systems, such as accessing host receptors, evading R/E systems, and anti-CRISPR proteins (Safari et al., 2020). However, if these defense systems in bacteria do not exist or work, change themselves through accelerated molecular evolution under phage infection stress should be another strategy. Conversely, phages can also generate mutation to increase their invasion ability. For instance, the rate of molecular evolution in phage φ2 was far higher when both *P. fluorescens* and the phage coevolved with each other than when phage evolved against a constant host genotype (Paterson et al., 2010). The most rapidly evolving phage genes under coevolution were those involved in host infection (Paterson et al., 2010). Continual natural selection for adaptation and counter-adaptation drive molecular evolution of interacting bacteria and their phages (Gómez and Buckling, 2011; Koskella and Brockhurst, 2014). In this study, the distribution profiles of SNVs in coevolved cell populations were discrepant at different development stages, and it implied that coevolved cell populations adopted different adaptation strategies to adapt to variable infection stress which may be caused by genetically changed phages.

At last, we should point out that though totally 80 PRM strains of *V. alginolyticus* were acquired mainly based on the morphologies of resistant colonies, similar morphologies of resistant colonies may harbor divergent mutants as some genetic changes cannot be exhibited by the differences of common phenotype characteristics. Therefore, genotyping by IRS-PCR was adopted to first screen the genetically variable PRM strains as the method is simple and time-saving. However, genotyping by IRS-PCR cannot show all genetic changes of tested strains due to the limitation of this method such as short amplification fragments and the selection of restriction endonucleases, and thus it is likely that some PRM strains that were not selected for subsequent analysis may also harbor many genetic variations.

In summary, *V. alginolyticus* PRM strains exhibited abundant genetic changes and genetic divergence under phage infection stress. Resequencing revealed that coevolved *V. alginolyticus* populations with phages had discrepant profiles of SNVs and InDels at three coevolved stages, while most detected SNVs and InDels occurred in the coevolved populations. Mutations were observed within rRNA and tRNA genes in the PRM strains and coevolved cell populations. Our results combining with other findings indicated that phage infection stress can boost mutation rates in *V. alginolyticus* genome and accelerate the genetic divergence, and thus it is likely one driving force for the bacterial genome evolution.

## Materials and Methods

### The Primary *V. alginolyticus* Strain E06333

*Vibrio alginolyticus* strain E06333 was isolated from marine fish, *Epinephelus coioides*, in 2006, in Guangdong province, China, and stored in our laboratory (Luo et al., 2018). The strain E06333 was cultured in LB plates and single colony was picked out for subsequent experiments.

### Enrichment of Mixed Phages Infecting *V. alginolyticus*

Wastewater samples were collected from an outfall of shrimp aquafarm in Maoming, Guangdong, China, and the water samples were used to enrich mixed phages that can infect and lyse *V. alginolyticus* E06333 cells. The samples were centrifuged at 10,000 *g* for 10 min, and then the supernatants were filtered using a sterile 0.45-μm filter. The filtrates were mixed with the same volume of 2 × LB broth and then were added with 1% overnight culture of *V. alginolyticus* E06333 cells followed by incubation at 30°C for 12 h. The lytic culture samples (which remained transparent) were centrifuged, the supernatant was pipetted out, and was then filtered through a sterile 0.22-μm filter. The filtrate was used as enriched phages and was stored at 4°C.

### Isolation of *V. alginolyticus* Phage-Resistant Mutant Strains From Phage-Resistant Colonies

The double-layer plates were used to isolate PRM colonies (Carlson, 2005). Briefly, each of 0.5-ml cultures of *V. alginolyticus* at *OD*_600 nm_ = 0.6 was mixed with 0.5 ml of abovementioned enriched phage fluid at an approximative multiplicity of infection (MOI) of 30, and added into 4-ml LB broth (supplemented with 0.7% agar) preheated at 45°C, and then immediately poured onto the lower LB agar plate. The plates were incubated overnight at 30°C and then PRM colonies with different morphologies were picked and restreaked for the isolation of individual colonies more than four times.

### Genotyping of *V. alginolyticus* Phage-Resistant Mutant Strains With Infrequent-Restriction-Site-PCR

The *Hha*I adaptors (AH1 and AH2) and the *Xba*I adaptors (*Xba*I-ad1 and AX2) were prepared as described previously (Ren et al., 2008). The Primer PN-X was constructed to complement *Xba*I-ad1. Sequences of the other four primers (PN-A, PN-T, PN-C, or PN-G) were identical to that of PN-X except that an additional base (A, T, C, or G, respectively) was placed at their 3’ ends. The digestion and ligation of template DNA were carried out as described previously (Mazurek et al., 1996). Less than 1 μg genomic DNA was digested with 10 U of *Hha*I (TaKaRa) and 10 U of *Xba*I (TaKaRa) in 1 × T + BSA buffer (final volume, 20 μl) for 2 h at 37°C. T4 DNA ligase (525 U), 10 × T4 buffer (3 μl), the *Xba*I adaptor (10 pmol), the *Hha*I adaptor (10 pmol), and water were added for a total volume of 20 μl. The mixture was incubated at 16°C for 1 h to ligate the adaptors to the digested DNA and then at 65°C for 20 min to inactivate T4 DNA ligase. The sample was digested with 5 U of *Xba*I and 5 U of *Hha*I at 37°C for 15 min to cleave any restriction sites reformed by ligation. Amplification was performed in the optimized conditions in which a 50 μl PCR mixture included 25 ul of PrimeSTAR Max Premix (2×), 5 μl of restricted–ligated DNA, 1 μl (10 μM) AH1 and 1 ul (10 μM) either PN-X, PN-A, PN-T, PN-C, or PN-G, then sterilized distilled water was added to 50 ul. Amplification condition consisted of an initial denaturation of 98°C for 3 min and 38 cycles of 10 s at 98°C, 5 s at 60°C, 30 s at 72°C and a final extension of 10 min at 72°C. All the enzymes were bought from TaKaRa Company (Japan). The sequences of primers and adaptors are shown in Supplementary Table 2. The PCR products were loaded into wells of a 8% polyacrylamide gel prepared from a 30% acrylamide–bisacrylamide (29:1) solution in 1 × TBE buffer (0.045 M Tris-borate, 0.001 M EDTA). After electrophoreses for 5 h at 200 V, the gel was stained with ethidium bromide (0.5 mg/ml) for 30–45 min, destained in water for 25 min, and photographed with UV illumination.

### The Analysis on Biochemical Profiles of Selected *V. alginolyticus* Phage-Resistant Mutant Strains

The GEN III microplates (BIOLOG, United States) assaying the utilization of 71 carbon sources and the sensitivity to 23 chemical substrates were adopted to exhibit the potential shift of biochemical profile in PRM strains singled out by IRS-PCR. The cells of tested strains were suspended in inoculating fluids, and the aliquots of 100-μl suspended cells were added into the microplates. After 24–36 of hours incubation, the microplates were analyzed by a Biolog Microstation System (Biolog, United States).

### Whole-Genome Sequencing of the Selected Phage-Resistant Mutant Strains

Genomic DNAs of PRM strains were sheared, then approximately 500-bp DNA fragments were selected. DNA fragments were end repaired and ligated with Illumina universal adapters. After adapter ligation, DNA fragments were further size-selected on an agarose gel and PCR-amplified for 6–8 cycles using the Illumina P1 and Index primer pair and Phusion^®^ High-Fidelity PCR Master Mix (NEB, China). The final library was purified using Agencourt AMPure XP beads and quality-assessed by Agilent Bioanalyzer 2100 (DNA 7500 kit) to determine library quantity and fragment size distribution before sequencing. The genomes of the selected PRM strains were *de novo* sequenced using the Illumina HiSeq2500 platform (GENEWIZ, China), and the sequencing coverage depth ranged from 385× to 420×. Reads by quality control were assembled using velvet (v1.2.10) (Zerbino and Birney, 2008; Zerbino et al., 2009) and gap-filled with SSPACE (v3.0) (Boetzer et al., 2011) and GapFiller (v1–10) (Boetzer and Pirovano, 2012). Genome comparison between the PRM strains and ancestor *V. alginolyticus* E06333 (GenBank: CP071058-CP071059) was carried out by MAUVE (Darling et al., 2004) software. The coding genes were annotated by Glimmer (v3.02) (Delcher et al., 2007). GO (Gene Ontology) database (Harris et al., 2004) and KEGG (Kyoto Encyclopedia of Genes and Genomes) database (Kanehisa and Goto, 2000) were used to annotate the functions of genes and pathways, respectively. Non-coding RNA genes were predicted by Rfam database (Nawrock et al., 2015).

### Evolved and Coevolved Cell Populations and Resequencing

*Vibrio alginolyticus* E06333 was cultured at the early exponential stage (*OD*_600 nm_ = 0.6–1), 5 ml of culture fluid was collected and labeled as sample T0. At the same time, 40 replicates of 5 ml LB broth were inoculated with 60 μl of *V. alginolyticus* E06333 culture fluid. Among them, 20 replicates were inoculated with 20 μl of phage fluid at a multiplicity of infection (MOI) about 10 to serve as coevolved populations, wherein bacterial cells coevolved with enriched phages, and the other 20 replicates were inoculated with 20 μl of LB broth without the addition of phages to serve as evolved populations. Coevolved and evolved populations were incubated at 30°C in a shaker.

When OD_600 nm_ of cultures in coevolved populations reached about 0.4, 1.0, and 1.5, five replicates in coevolved populations were mixed and labeled as samples VE1, VE2, and VE3, respectively, and at the same timepoints five replicates in evolved populations were mixed and labeled as samples VC1, VC2, and VC3, respectively. The cells of all the samples were centrifuged and collected for DNA extraction.

Genome resequencing of *V. alginolyticus* cell populations from all the samples was carried out by Illumina HiSeq × 10 and the sequencing coverage depth for each base site exceeded 230× (most exceed 400×) Pass filter data were removed adaptors and bases of low quality by Cutadapt (v1.9.1) to obtain clean data for continuous data analysis. Alignment software BWA (v0.7.12) (Li and Durbin, 2009) was used to map clean data to the reference genome of *V. alginolyticus* E06333. The detection of SNV (single base variation) and InDel (insert or deletion mutation) was performed using Samtools (v1.1) (Li et al., 2009) and the Unified Genotyper module of GATK (v3.4.6) software (DePristo et al., 2011). Annotation for SNV/InDel was performed by Annovar (Wang et al., 2010). Besides, InDels were detected and their frequencies were calculated no matter how many bases they consisted. Considering the accuracy of resequencing, only the mutation frequencies >10% were counted.

## Data Availability Statement

The datasets presented in this study can be found in online repositories. The names of the repository/repositories and accession number(s) can be found below: https://www.ncbi.nlm.nih.gov/genbank/, CP071058; https://www.ncbi.nlm.nih.gov/genbank/, CP071059.

## Author Contributions

All authors contributed to the writing of the manuscript. PL and YA conceptualized and designed the experiments. WZ and YL performed the experiments including IRS-PCR, Biolog assays, and DNA extraction and carried out bioinformatic analysis. ZL isolated PRM strains. XJ isolated the phages infecting *V. alginolyticus*. CH reviewed the manuscript and offered partial funding. PL and WZ wrote the manuscript.

## Conflict of Interest

The authors declare that the research was conducted in the absence of any commercial or financial relationships that could be construed as a potential conflict of interest.

## Publisher’s Note

All claims expressed in this article are solely those of the authors and do not necessarily represent those of their affiliated organizations, or those of the publisher, the editors and the reviewers. Any product that may be evaluated in this article, or claim that may be made by its manufacturer, is not guaranteed or endorsed by the publisher.
