# Supplementary material for: Genomic Changes and Genetic Divergence of Vibrio alginolyticus Under Phage Infection Stress Revealed by Whole-Genome Sequencing and Resequencing
Source: Front Microbiol. 2021 Oct 4;12:710262. doi: 10.3389/fmicb.2021.710262 (PMC8521149; doi:10.3389/fmicb.2021.710262)
Supplement: Supplementary file 1 [file Data_Sheet_1.zip › Table S2.docx]

Table S2 The sequences of primers and adaptors used in this study

| Primers or adaptors | Sequences (5’ to 3’) |
| --- | --- |
| AH1 | AGA ACT GAC CTC GAC TCG CAC G |
| AH2 | TGC GAG T |
| XbaI-ad1 | PO_4_ -CTA GTA CTG GCA GAC TCT |
| AX2 | GCC AGT A |
| PX | AGA GTC TGC CAG TAC TAG A |
| PX-G | AGA GTC TGC CAG TAC TAG AG |
| PX-C | AGA GTC TGC CAG TAC TAG AC |
| PX-T | AGA GTC TGC CAG TAC TAG AT |
| PX-A | AGA GTC TGC CAG TAC TAG AA |
